# On the Treatment of Acute Rheumatism

**Published:** 1850-11

**Authors:** 


*On the Treatment of Acute Rheumatism.* By GEO. L. UPSHUR, M. D., Norfolk, Va.

A celebrated physician being once asked what he considered the most successful treatment of acute rheumatism, answered, "six weeks," thereby plainly intimating his opinion of the intractable nature of the disease. While I am ready to admit that there is *some* truth in this opinion, and that rheumatism now and then assumes a form which might well entitle it to a place among the *opprobria medicorum,* I am not at all prepared to entertain the notion that early and judicious treatment has no power, in a majority of cases, to alleviate its pangs, or shorten its duration. On the contrary, I believe that rheumatism is as amenable to proper management as any disease of equal severity.

Within the past five years, I have accurately noted the history and treatment of about *thirty* cases of acute articular rheumatism, from among which the twelve cases just detailed are indiscriminately taken.* In these, it will be seen, the average duration of the disease was 5½ days; the longest being 14, and the shortest 2 days. *Croton oil* was used in 11, *colchicum* in 6, *quinine* in 6, *blood-letting* in 2, and *iodide of potassium* in 2. I desire to say a word or two upon each of these agents.

Of *Croton oil* as a purgative in acute rheumatism, I am prepared to speak in the highest terms. Cathartics have always stood in the front rank of remedies in this disease, but I am disposed to believe that the efficacy of Croton oil does not depend entirely upon its cathartic properties; it possesses a power over the disease beyond these, and apparently not dependent upon them, for other cathartics, which act as powerfully and as promptly, producing similar watery stools, do not bring a like amount of relief to the patient. I do not say that it is a *specific,* for I am not a be-

* The cases are omitted.—EDITOR BUFFALO MEDICAL JOURNAL.

liever in the doctrine of specifics, in medicine; that doctrine has put more stumbling blocks in the way of medical progress than all the open quackery of the past half century. I merely desire to state, that after a fair trial, in a number of cases accurately observed, where there was scarcely a possibility of falling into error, I believe that the Croton oil is the *best* single remedy in the treatment of acute rheumatism; and I am thoroughly convinced, that it is as justly entitled to the term *specific*, in this disease, as is quinine in miasmatic fever. I speak now, not of chronic rheumatism, nor of that which results from a syphilitic taint, but of the acute inflammatory affection. In all the eleven cases, in which it was given, its beneficial effect was palpably evident, but particularly as in cases 5, 6, 8, 10, 11, and 12.

Dr. Wm. A. Thom, an intelligent physician of Northampton County, to whom I am indebted for the first hint in regard to the value of Croton oil in this disease, informed me, about two years ago, that he had used it in several severe cases, and uniformly with success.

*Colchicum* has uniformly disappointed me. In cases 1, 4, 7, it was fairly tried, and although it invariably acted freely upon the bowels, there was no alleviation of the distressing symptoms. In the chronic disease, I think I have sometimes derived great benefit from it, but in the acute form, *never*. It may be that I have been so unfortunate as always to get hold of an inferior preparation, but some how or other, I have taken the notion, that when a patient is treated with colchicum *only*, he would do just as well to take old Dr. Warren's *six weeks* instead.

*Quinine* I would place next to Croton oil in the treatment of rheumatism. That it is decidedly *sedative* in large doses, is undeniable, and therefore it may be exhibited during the febrile stage. Given after the use of the lancet, in highly inflammatory cases, or after powerful purgation with Croton oil, it has been productive of the happiest effects in my hands. I never give to an adult a smaller dose than ten grains, repeated three or four times a day; less than this would be worse than useless. It is particularly serviceable in those cases which perspire most profusely.

It seems to me that *blood-letting* possesses no direct *curative* power in the treatment of rheumatism. In robust persons, with high fever, intense pain, and a full, bounding pulse, I think it subserves a useful purpose in rapidly subduing the over-excited circulation, thus placing the system in a better condition to respond to the impression of true curative agents. It is true that blood-letting is one of the most powerful antiphlogistics, and that rheumatism, in its acute form, presents undeniable evidences of its highly inflammatory character, nevertheless it has long since been voted an inflammation, *sui generis,* and I believe there is a great deal too much of the *neuralgie* about it to justify the heroic use of the lancet, so warmly advocated by Bouillaud. *Topical* depletion has never been of the least service in my hands, except where the disease was confined to a single joint, *with no disposition toward metastasis.*

When the disease assumes the chronic form, or if there should exist a scrofulous, or syphilitic taint, no remedy will be found equal to the *iodide of potassium.* I usually order five grains four times a day, to be taken in hop tea, the bowels in the meantime being opened every day with the black draught, or other cathartic. I have rarely seen the most obstinate cases refuse to yield to this treatment.

In the second attack of Case 7, almost immediate relief followed copious purgation with the decoction of *may-apple,* (podophyllum,) but it is very unphilosophical, particularly in so uncertain a science as Therapeutics, to deduce a general conclusion from a solitary observation, so I will not venture to recommend the may-apple until authorized by further trial. I have adverted to it, merely because it forms a part of the history of the case; and with the remark that it is considered by all of the old *negro-doctors* in this section of country as a *specific* in rheumatism, I dismiss the subject.—*Medical Examiner.*

